# Adhesion of Resin to Lithium Disilicate with Different Surface Treatments before and after Salivary Contamination—An In-Vitro Study

**DOI:** 10.3390/bioengineering9070286

**Published:** 2022-06-29

**Authors:** Ryan Harouny, Louis Hardan, Elie Harouny, Cynthia Kassis, Rim Bourgi, Monika Lukomska-Szymanska, Naji Kharouf, Vincent Ball, Carlos Khairallah

**Affiliations:** 1 Department of Restorative Dentistry, School of Dentistry, Saint-Joseph University, Beirut 1107 2180, Lebanon; ryaneliott.harouny@net.usj.edu.lb (R.H.); louis.hardan@usj.edu.lb (L.H.); elie.harouny@usj.edu.lb (E.H.); cynthia.kassis@usj.edu.lb (C.K.); rim.bourgi@net.usj.edu.lb (R.B.); carlos.khairallah@usj.edu.lb (C.K.); 2 Craniofacial Research Laboratory, Division of Biomaterials, School of Dentistry, Saint-Joseph University, Beirut 1107 2180, Lebanon; 3 Department of General Dentistry, Medical University of Lodz, 251 Pomorska St., 92-213 Lodz, Poland; monika.lukomska-szymanska@umed.lodz.pl; 4 Department of Endodontics, Faculty of Dental Medicine, Strasbourg University, 67000 Strasbourg, France; dentistenajikharouf@gmail.com; 5 Department of Biomaterials and Bioengineering, INSERM UMR_S 1121, Strasbourg University, 67000 Strasbourg, France

**Keywords:** decontamination, lithium disilicate, resin, saliva, shear bond strength

## Abstract

The salivary contamination occurring at the try-in procedures of lithium disilicate (LDS) can jeopardize their bond strength. Various laboratory reports have concluded that applying 37% phosphoric acid (H_3_PO_4_) could be considered as a predictable way of removing salivary contaminants. An experimental method that consists of sealing the intaglio of the ceramic restorations with a layer of cured adhesive could allow consequent time saving for dental practitioners. It is, besides, necessary to establish an optimal decontamination protocol. Hence, this study aimed to determine the most efficient surface treatment, before and after salivary contamination, by comparing the adhesion between resin and LDS. In order to do so, five groups of ten specimens (*n* = 10) each underwent the different types of surface treatments before bonding, followed by 2500 cycles in the thermocycler. A shear bond strength (SBS) test was then conducted on a universal testing machine (YLE GmbH Waldstraße Bad König, Germany), followed by a fracture-type analysis on an optical microscope (Olympus BX53, Shinjuku, Tokyo, Japan). Statistical analysis was set with a level of significance of α = 0.05. The surface treatment significantly affected the SBS results. The decontamination with HF (12.59 ± 2.71 MPa) and H_3_PO_4_ (13.11 ± 1.03 MPa) obtained the highest values, silanizing only before contamination obtained intermediate values (11.74 ± 3.49 MPa), and silanizing both before and after the salivary contamination (10.41 ± 2.75 MPa) along with applying a bonding agent before contamination (9.65 ± 1.99 MPa) resulted in the lowest values. In conclusion, H_3_PO_4_ proved to be efficient, thus, allowing the practitioner to avoid the clinical use of HF; it can, therefore, be considered as a valid alternative. Presilanization and resilanization of specimens, along with applying a bonding agent before contamination, did not yield satisfying results.

## 1. Introduction

All-ceramic restorations, including lithium disilicate (LDS), are increasingly preferred by both patients and practitioners [1]. With their esthetic advantage and their satisfying durability, they represent a good alternative to conventional ceramo-metallic crowns [2,3]. An important step for this success is proper bonding [4], which includes etching the intaglio with HF and then applying a silane agent. Furthermore, it is essential to find the best way to clean the prosthetic intaglio of the saliva after the intra-oral try-in, since this thin film is known to reduce the bond strength [5,6]. Numerous techniques have been proposed and tested, such as cleaning with ethanol, isopropanol, sodium hypochlorite, water spray, or putting the restoration in an ultrasonic bath, but the results were not satisfying [5,7,8,9]. Although the standard method is to conduct the try-in of the restoration in the mouth before applying the HF and silane agent, the etching of the restoration is sometimes undergone at the dental laboratory [9]. This is either done to make it easier for the practitioner or because chairside use of HF is prohibited in some countries due to its potential hazardous effects [10,11,12,13,14]. It is, therefore, necessary to have an efficient alternative to HF in order to clean the intaglio after the try-in. This could be made possible by using phosphoric acid (H_3_PO_4_), which is considered an interesting cleaning method due to its low cost and availability in every practitioner’s clinic. It yields the same findings as non-salivary contaminated LDS specimens according to a previous study [15], or slightly or significantly lower bond strength according to other studies [5,9,16]. This disparity of results made the inclusion of H_3_PO_4_ in this study interesting.

Aside from the cleaning methods applied after the try-in, it also seems valuable to evaluate the influence of the intaglio’s pretreatment on the bond strength obtained after contamination and cleaning.

Silanizing the specimens after the HF etching and before their contamination yielded a significant increase in the bond strength obtained compared to specimens without pre-silanization and treated with the same decontamination method. Nevertheless, only a few studies took this parameter into account. It is worth noting that this technique goes against the manufacturer’s recommendations, which warns of eventual harm to the silane layer [7,17].

Moreover, the need to resilanize the specimens, which were pre-silanized before contamination, should be evaluated. This parameter was, therefore, included in this study.

Lastly, an experimental method, which is already used in some countries, could help to avoid the step of HF etching in dental practice while still protecting the etched prosthetic intaglio and the silane layer by applying and polymerizing an adhesive layer on the intaglio after the etching and the silanization, but before the try-in. After trying the restoration in the mouth, the intaglio would simply be cleaned with ethanol and the adhesive surface simply reactivated by a new adhesive layer. However, only one study describing this method is available [17]. It was consequently included in this manuscript, with an additional innovation, by applying this method on a more frequently used material, LDS, on which it had not been tested before. LDS was chosen for being a dental biomaterial well known by practitioners, as they frequently use it when high aesthetic is requested by the patient. LDS has a high translucency when compared to the opacity shown by zirconia, and it also presents high mechanical properties and good long-term survival rates [18,19,20].

Hence, the main objective of this study was to evaluate the effect of different pre-treatments and cleaning methods following contamination by saliva on the adhesion of resin to LDS after ageing. The null hypothesis was that the surface treatment before and after the contamination would not have a significant influence on the bond strength of resin to LDS between the different groups.

## 2. Materials and Methods

### 2.1. Specimen Preparation

LDS blocks (*n* = 50) were used after the approval of the Institutional Review Board of Saint-Joseph University (FMD-SF30; ref.#USJ-2020-163).

Type, brand, composition, lot number, and manufacturer of the materials used in this study are listed in Table 1.

LDS blocks were cut before crystallization with a low-speed precision cutting machine (Exakt 30, EXAKT Vertriebs GmbH, Norderstedt, Gemany) to obtain 50 specimens with the following dimensions: 5 mm length, 5 mm width, and 3 mm height. The specimens were then crystallized according to the manufacturer’s instructions and embedded in acrylic resin (Novacryl, Tricodent LTD, Victoria Road, Burgess Hill, England), then poured in Ultradent’s plastic mold. The exposed bonding surface was polished with 600 μm grit silicon carbide paper under irrigation for one minute to obtain a flat surface. The specimens were thereafter placed in an ultrasonic bath with distilled water for five minutes and subsequently dried with an air syringe. They were then randomly divided into five groups of ten specimens each, according to the surface treatment to be performed.

**Figure 1:**
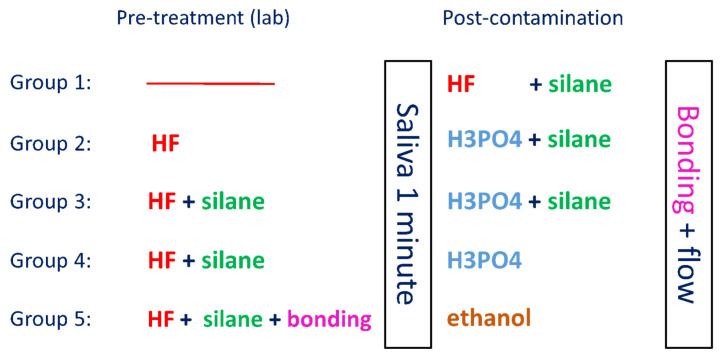
Surface treatment methods of the different groups (hydrofluoric acid (HF); phosphoric acid (H_3_PO_4_)).

All specimens were treated by a single operator and with saliva freshly collected the same day. Figure 1 shows the surface treatment methods executed in the different groups.

### 2.2. Bonding of the Specimens

After surface treatment, all the specimens received a layer of adhesive resin (Adper Single Bond 2, 3M ESPE) spread with a gentle air stream for 15 s to evaporate the solvent [21,22] and to obtain a homogeneous thickness, then polymerized for 20 s (Woodpecker 1000–1200 mW/cm^2^). A polyethylene cylindrical mold with an internal diameter of 2.38 mm and a height of 2.15 mm (Bonding Jig, Ultradent Products, Inc., South Jordan, UT, USA) [23,24] was then placed on the surface of the specimens covered with the adhesive, and flowable composite (Filtek Z350 XT Flowable Restorative, A1 Shade, 3M ESPE) was injected in it from the bottom to the top, with the tip of the syringe kept inside the material to avoid the incorporation of air bubbles. The flowable resin was then polymerized for 40 s with the same light-curing device [8,9].

The bonded specimens were washed with an air-water spray and kept for 24 h in distilled water at 37 °C, in an incubator, to allow complete polymerization of the resin [25].

### 2.3. Bond Strength Test and Failure Analysis

After thermocycling (2500 cycles, 5–55 °C, dwell time: 45 s, transition time: 15 s), the shear bond strength (SBS) tests for all the specimens were performed on a universal testing machine (YLE GmbH Waldstraße Bad König, Germany) with a knife-edge blade placed perpendicularly and touching only the bonding interface, at a crosshead speed of 1 mm/min until fracture occurred, according to ISO/TS 11405:2015 [26]. The SBS was then calculated according to the following formula: R = F/A (R being the bond strength in MPa, F the failure force in Newtons, and A the bonding area in mm^2^).

After debonding, the type of fracture was determined under x10 magnification with an optical microscope (Olympus BX53, Shinjuku, Tokyo, Japan) and classified as follows:

(a) Either adhesive: between the ceramic and the resin; no remnant of composite resin on the ceramic surface; (b) either cohesive: within the composite resin; resin remnants can be seen on most of the ceramic surface; (c) either mixed (combination of adhesive and cohesive failure): remnants can be seen on parts of the ceramic, while other parts have no remnant.

### 2.4. Statistical Analysis

IBM SPSS Statistics version 26.0 was used to analyze the data. The level of significance was set at -*p*-value ≤ 0.05. The primary outcome measurement was the SBS (MPa). Kolmogorov–Smirnov tests were used to assess the normality of the distribution of the variables. Levene’s test was used to assess the homogeneity of variances between groups. One-way analysis of variance (ANOVA) followed by Tukey (HSD) post-hoc tests were used to compare mean bond strength between groups. Fisher Exact tests were used to compare the type of fracture among groups.

## 3. Results

### 3.1. Shear Bond Strength Results

The SBS was significantly different between the five groups (-*p*-value = 0.019; ANOVA).

**Figure 2:**
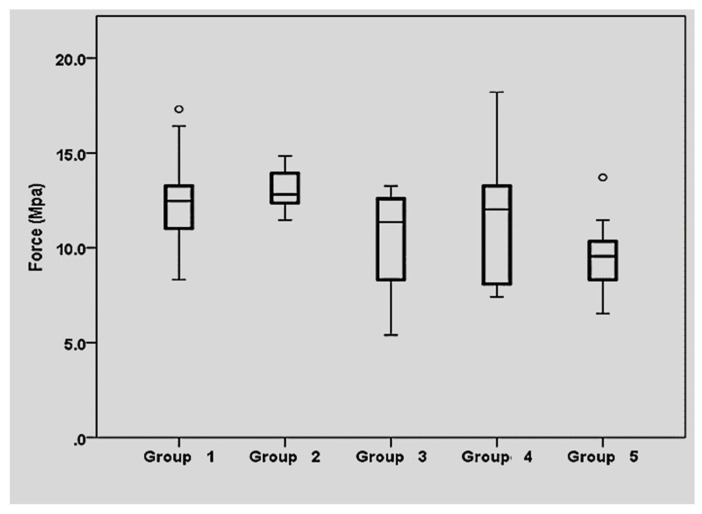
Mean shear bond strength of the different groups. Surface treatment: Group 1: Cleaning with HF after contamination; Group 2: cleaning with H_3_PO_4_ after contamination; Group 3: Silanization before and after contamination; Group 4: Silanization only before contamination; Group 5: Sealing of the ceramic with adhesive. °: Extreme outliers: the outliers are points that stay out of the interval.

The highest mean SBS was obtained by groups 1 (12.59 ± 2.71 MPa) and 2 (13.11 ± 1.03 MPa) (Figure 2). Group 4 showed intermediate values (11.74 ± 3.49 MPa) while the lowest results were obtained in groups 5 (9.65 ± 1.99 MPa) and 3 (10.41 ± 2.75 MPa). The SBS values obtained are listed in Table 2, along with the mean, the standard deviation, the 95% confidence interval, and the minimal and maximal value.

### 3.2. Comparison of the Types of Fracture

**Figure 3:**
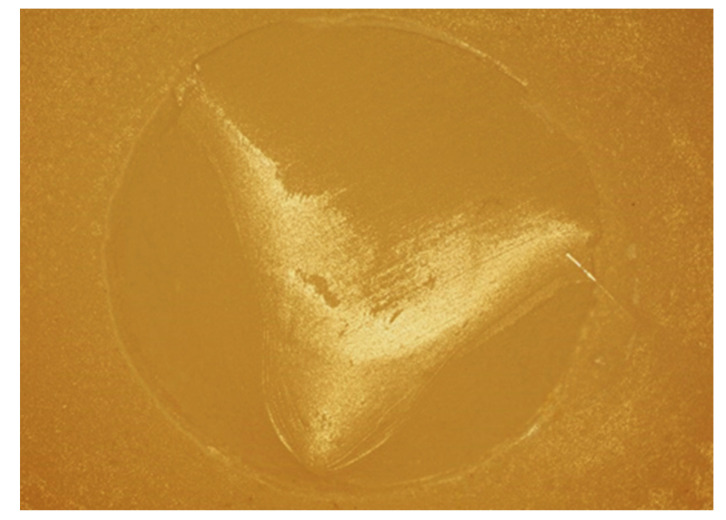
Representative image (optical microscope x10) of a mixed failure.

**Figure 4:**
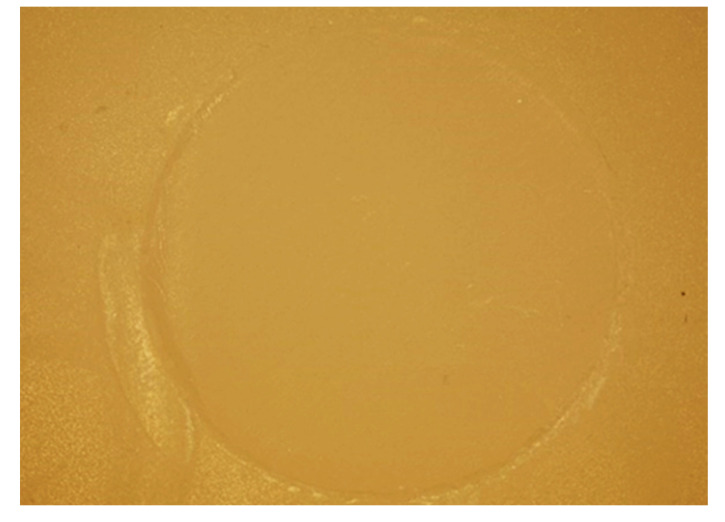
Representative image (optical microscope x10) of an adhesive failure.

Cuts obtained with an optical microscope (Olympus BX53, Shinjuku, Tokyo, Japan) (x10) show the aspect of adhesive and mixed fractures (Figure 3 and Figure 4).

**Figure 5:**
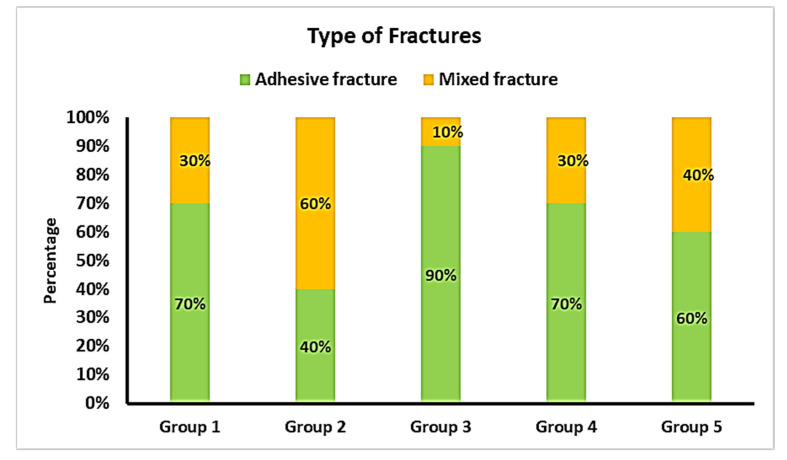
Distribution of the types of fractures in the different groups.

Figure 5 shows the distribution of the types of fractures among the groups tested.

## 4. Discussion

This study was conducted to establish the most efficient surface treatment before and after salivary contamination of LDS specimens, in order to ensure an optimal bond strength of resin to ceramic after artificial aging. According to the results obtained, the mean SBS was significantly different depending on the surface treatment. Therefore, the null hypothesis tested in this study was rejected.

Many contaminants can impair the bonding of ceramic restorations, such as blood, silicone, dental stone, or isolation medium. However, saliva remains the most relevant from a clinical point of view. The adhesion of saliva to restorations and to the surface of the teeth leads to the formation of a thin pellicle that reaches a thickness of 10 to 20 nm within a few minutes [6]. This layer is not eliminated by water rinsing [16] and has a negative influence on the wettability and surface free energy of the substrate [9]. An inefficient decontamination could cause an important decrease in bond strength values, as shown in numerous studies, and saliva should, therefore, be correctly eliminated in order to achieve a long-lasting adhesion [9,27,28].

The specimens in groups 1 and 2 showed the best results. Group 1, where the ceramic was etched with HF after contamination, was the control group, since it represents the typical situation of a practitioner decontaminating ceramic restorations after the try-in following the universally accepted protocol. The specimens in group 2 were decontaminated with H_3_PO_4_. The high values obtained with the first method correspond with those obtained in other studies [9,15,16,27,28,29], where values after HF decontamination were similar to the control (uncontaminated) group. The results of the second method were more controversial in the literature; they were identical to the control group on LDS [15], on feldspathic ceramic [30], and on zirconia [31], like in this study, unlike other studies, where it was either fair [5,16] or unsatisfying [28]. Although it is not entirely clear how phosphoric acid helps in removing saliva, it is suggested that it perhaps penetrates the salivary film and then etches the surface of the ceramic below it, liberating the saliva from the surface [6].

Specimens in groups 3 and 4 received a layer of silane before contamination. Abboush et al. and Marfenko et al. reported that this would increase the bond strength [17,30], while Alfaro et al. did not obtain better results with this method [16].

Group 4, silanized only before contamination, yielded intermediate values, which were significantly lower than those in groups 1 and 2. This could be due to the fact that the silane layer was put in one week prior to contamination, decontamination, and bonding, to simulate the time of delivery from the laboratory. The mechanical application of saliva with a microbrush and the eventual degradation by H_3_PO_4_ could be added as factors that could have harmed the silane layer, therefore, decreasing the values obtained. Marfenko et al. questioned the stability of the silane layer after a mechanical action, although their study addressed mechanical cleaning with Ivoclean [17].

Group 3, however, in which silanization was performed before and after contamination, showed values significantly lower than those in group 4, and statistically similar to those in group 5. This could be explained by the negative impact of the application of thick or multiple silane layers, which could interact with one another and interfere with the bonding of the resin to the ceramic [7]. It is, therefore, possible that the application of a second layer of silane could have affected the bonding more than the application of H_3_PO_4_ on the first layer of silane, since H_3_PO_4_ acted chemically, not mechanically, which yielded significantly higher values in group 4 compared to group 3. However, these results are in contradiction with those of other studies [7,17,30], which did not show a deleterious impact to reapplying silane after decontamination. Further research is, therefore, deemed necessary to clarify the effect of such a procedure.

Group 5, which represented the experimental method of this study, with the sealing of the ceramic with a bonding agent before contamination, obtained the lowest values, unlike the study of Bomicke et al. [27], which showed that after thermocycling, values are significantly higher than those in the uncontaminated group. Numerous factors that differ between these two studies could explain the heterogeneity of the results: the material on which the bonding was applied (LDS compared to lithium silicate reinforced with zirconia), the bonding agent, the bond strength test setting (SBS test compared to tensile bond strength test), the method used for artificial aging (2500 thermal cycles versus 6 months of storage in distilled water), and the diameter of the composite resin (2.38 mm versus 3.3 mm). It is important to note that in the study of Bomicke et al., pre-polymerized “core build-up” composite resin cylinders were bonded to the ceramic with a resin cement, which differs from the application of the flowable resin on the ceramic topped with a layer of polymerized adhesive, as was performed in this study. To our knowledge, these two studies are the only ones that evaluated this method and further research is, therefore, necessary to make a conclusion about its efficiency.

Yet, the quality of the bonding should not only be evaluated by bond strength values. The type of fracture (adhesive, cohesive, mixed), determined with a microscope, is also an indicator of the bonding mechanism [15,32]. A higher incidence of adhesive fractures indicates a lower quality of bonding in one group of specimens when compared to others.

The specimens in group 2, which had the highest mean SBS value (13.11 ± 1.03 MPa), showed the most mixed fractures (60%), while the specimens in group 3, which had significantly lower mean SBS values (10.41 ± 2.75 MPa), showed only 10% mixed fractures. However, the difference in the type of fracture rate was not statistically significant, which does not allow any conclusion. This is probably due to the relatively small number of specimens, which is one of the limitations of the study. Nevertheless, it can be stated that the significant overall proportion of mixed failures is due to the testing method, namely the macroshear bond strength test. In fact, a wider bonding interface probably contains more defects [33,34], which raises the prevalence of cohesive and mixed failures when compared to the microtensile bond strength test. The latter, indeed, allows for a higher precision because of a more homogeneous distribution of the forces on the bonded interface [35,36,37,38,39], and it can, therefore, be considered as another limitation of this study.

Even so, the SBS test was chosen for its easy use, and it is important to mention that shear forces outweigh tensile forces on ceramic veneers. Studying this parameter would then be more interesting for this type of restoration where the absence of mechanical retention makes bonding an essential element for its longevity [40]. Moreover, when compared to a microtensile bond strength test, it helps avoiding pre-testing failures since it does not need to be cut before testing, which would be problematic with a brittle material, such as ceramic [6,17].

This research did not include a group of specimens without saliva contamination, since it has already been proved that etching with HF allows one to obtain the same bond strength values as before contamination [9,15,28], so these two groups would lead to identical values.

Similar to Lapinska et al. [9], this study used a flowable composite resin instead of a resin cement, for two reasons. Firstly, the principal aim of the study was not to evaluate the resistance of resin itself but the surface treatment of LDS and its influence on the bond strength values. Secondly, these two types of materials share the same physical and chemical properties because of their resin matrix and their comparable filler content. It is, consequently, possible to use one instead of the other in some circumstances.

Further, all the specimens underwent thermocycling, since the long-term values are the most relevant to the practitioner. This was also the case in several studies [6,17,28], where all the specimens were subjected to thermocycling, which was considered more suitable than water storage [25,41] to simulate the aging of the restorations and their bonding to resin.

Although decontamination with H_3_PO_4_ prevents the chairside use of HF, it does not change the fact that the protocol of decontamination and bonding after the try-in is time-consuming. Future studies on LDS and with a higher number of specimens should be undertaken, along with the method evaluated by Bomicke et al. [27], which is the sealing of the ceramic before contamination, because any efficient method that can save chairside time would be advantageous for both the practitioner and the patient.

## 5. Conclusions

According to the results obtained in this study, it can be concluded that H_3_PO_4_ is as efficient in removing saliva contamination from the surface of LDS as HF. On the other hand, the silanization only before contamination lessened bond strength when compared to the specimens that were not silanized prior to contamination. Resilanizing of pre-silanized specimens that were cleaned with H_3_PO_4_ did not improve bonding to LDS. The experimental method consisting of sealing the intaglio with a layer of cured adhesive did not prove its efficiency. Therefore, none of the pre-treatment methods tested allowed superior bond strength of resin to LDS.

## Figures and Tables

**Table 1:** Specifications of the materials used in the study.

Material	Brand	Lot	Composition	Manufacturer
Glass-based ceramic	IPS e.max CAD LT A1 shade	Y30837	SiO_2_,LiO_2_, K_2_O, P_2_O_5_, ZrO_2_, ZnO, other oxides, coloring oxides	IvoclarVivadent, Schaan, Liechtenstein
Ceramic etchant	Porcelain Etch	BGTV7	9% buffered hydrofluoric acid	Ultradent, Schaan, Liechtenstein
Ceramic primer	Porcelain Primer	1900001117	Pre-hydrolyzed silane primer with alcohol and acetone	Bisco, Schaumburg, IL, USA
Etching gel	DentoEtch	DE-4.12	37% phosphoric acid	Itena, Avenue Foch, Paris, France
Bonding agent	Adper Single Bond 2	NA61948	Bis-GMA, HEMA, dimethacrylates, ethanol, water, photoinitiators, methacrylate functional copolymer of polyacrylic and polyitaconic acids, and silica nanofiller	3M ESPE, St. Paul, MN, USA
Flowable composite	Filtek Z350 XT, Flowable Restorative, A1 shade	NA37278	Bis-GMA, TEGDMA, procrylatresins; ytterbium trifluoride, silica, zirconia/silica cluster fillers	3M ESPE, St. Paul, MN, USA

Silicon dioxide (SiO_2_), Lithium superoxide (LiO_2_), Potassium oxide (K_2_O), Phosphorus pentoxide (P_2_O_5_), Zirconium dioxide (ZrO_2_), Zinc oxide (ZnO), Bisphenol A-glycidyl methacrylate (Bis-GMA), Hydroxyethylmethacrylate (HEMA), Triethylenglycol-di-methacrylate (TEGDMA).

**Table 2:** Shear bond strength values. Groups with the same letter are not significantly different (*p* > 0.05).

	N	Mean	Standard Deviation	95% Confidence Interval		Minimum	Maximum
				Lowerbound	Upperbound		
Group 1	10	12.59 ^a^	2.71	10.65	14.53	8.32	17.31
Group 2	10	13.11 ^a^	1.03	12.37	13.84	11.47	14.84
Group 3	10	10.41 ^b^	2.75	8.44	12.38	5.40	13.26
Group 4	10	11.74 ^a,b^	3.49	9.24	14.23	7.42	18.21
Group 5	10	9.65 ^b^	1.99	8.22	11.07	6.52	13.71
